# Construct validity and reliability of the Talent Development Environment Questionnaire in Caribbean youth track and field athletes

**DOI:** 10.1371/journal.pone.0227815

**Published:** 2020-01-24

**Authors:** Candice E. Thomas, Gavin Abbott, Paul B. Gastin, Luana C. Main

**Affiliations:** 1 Centre for Sport Research, School of Exercise and Nutrition Sciences, Deakin University, Geelong, Australia; 2 Institute of Physical Activity and Nutrition, School of Exercise and Nutrition Sciences, Deakin University, Geelong, Australia; 3 La Trobe Sport and Exercise Medicine Research Centre, School of Allied Health, Human Services and Sport, La Trobe University, Melbourne, Australia; Universidad de la Republica, URUGUAY

## Abstract

Caribbean nations stand to benefit significantly from the potential insights that can be gained from the assessment of their athlete talent development environments; which in turn can lead to the formulation of evidence-based strategies and improvements to their sport development pathways. The principal aim of this study was to examine the psychometric properties of the 25-item TDEQ-5 to determine its validity to assess the development environments of talented youth track and field athletes from six English-speaking Caribbean countries. As a secondary aim, we sought to examine athletes’ perceptions of their talent development environment within this context. Confirmatory factor analysis revealed adequate model fit of a re-specified model and good overall internal reliability of the scale, therefore offering support for its use within this context. Furthermore, adequate construct validity and internal reliability was found within three subscales (i.e., communication, holistic quality preparation and support network) with subpar scores within two subscales (i.e., long-term development focus and alignment of expectations). Preliminary findings on athletes’ perceptions of their environment revealed key strengths in coaches’ long-term development focus and communication, however deficiencies were noted in the accessibility and availability of sport-related support and preparation of athletes. In conclusion, the re-specified TDEQ-5 with 25 items appears to be a reliable and valid measure within the Caribbean context. However, it is recommended that the scale be used with some caution with regard to the interpretation of results for the ‘long-term development focus’ and ‘alignment of expectations’ subscales.

## Introduction

In spite of the many successes attained by Caribbean track and field athletes on the world stage, anecdotal reports suggest a high attrition and poor transition of young talented athletes within the sport [1], which is consistent with the drop out trends in some other nations [2–4]. Furthermore, research has predominantly focused on the hypothesized genetic predisposition of Caribbean athletes of West African descent for the sprint events [5]. Whereas genetics might be one explanation for athletic prowess [5], the impact of the developmental environment within which an athlete is nurtured plays a significant role on their long-term engagement and progression within the sport [6, 7]. Historically, within the Commonwealth Caribbean, economic development has been equated with sectors such as mineral extraction, agriculture and tourism [8]. Consequently, investment, policy formulation and planning in sport at the state and private sector levels have not been prioritized [9]. As such, there still exists significant underdevelopment in sporting structures, financing, sport governance and sport professionalization within the Caribbean region which adversely affects the development of young talented athletes [8, 9]. Nonetheless, the impact of the development environment on young athlete’s athletic engagement and progression within the sport remains to be investigated within the Caribbean context.

In a bid to achieve sporting excellence, investment levels in advanced sporting nations have been greater than ever in sporting systems and structures to identify and develop exceptionally talented athletes [10]. However, the successes attained by elite track and field athletes within the Caribbean region (e.g., Usain Bolt) have seemingly come in spite of the aforementioned limitations within the athlete development pathway rather than as a result of the implementation of effective talent development strategies. A conducive learning environment in which young athletes are given the opportunity to realize and nurture their potential is considered ideal [11]. For this reason, recent research has placed greater emphasis on understanding the talent development environments of young athletes and the way these shape, challenge and support developing talent. It is therefore important to increase the knowledge and understanding around what effective talent development entails, and how it can be applied in varied contexts. Martindale and colleagues introduced a quantitative approach towards examining the TDE of young athletes with the formulation of the talent development environment questionnaire (TDEQ) [12]. However, the scale has only been validated within athlete populations of more developed sporting nations.

The TDEQ was designed and developed in response to the growing need to understand and measure effective talent development processes as well as to facilitate a more evidence-based approach towards research in talent development [12, 13]. The factor structure of the TDEQ was initially examined through an exploratory factor analysis using 590 talented adolescent athletes from within the United Kingdom. The analysis generated a 59-item seven-factor structure (long-term development focus, quality preparation, communication, understanding the athlete, support network, challenging and supportive environment, and long-term developmental fundamentals). In recent years, the TDEQ seven-factor structure has undergone significant revisions both at the subscale and item levels in an attempt to address some of its reported psychometric limitations (i.e., low internal reliability, conceptual overlap of subscales, item overload on single a domain) [12, 14]. Wang, Sproule [15] later modified the seven-factor structure into a 36-item six-factor scale, however the factorial validity of the revised scale was not assessed and also contained issues of low internal reliability. The factor structure of the modified TDEQ-6 was later examined through both exploratory and confirmatory factor analysis [14]. The analysis yielded the current 25-item, five-factor structure (i.e., TDEQ-5) with the following subscales included as constructs: 1) long-term development focus (LTF); 2) alignment of expectations (AOE); 3) communication (COM); 4) holistic quality preparation (HQP); and 5) support network (SN).

The revised 25-item five factor structure (TDEQ-5) was validated within talented youth athletes of Singapore [14]. The authors concluded that the refined TDEQ-5 appeared to be a more practical, reliable and valid scale for measuring the key identified talent development environmental factors. However, further examination of the psychometric properties of TDEQ-5 in different contexts to generalize findings was recommended [14]. Consequently, great interest has been shown by researchers in the refinement and/or translation of the instrument to adapt to specific athlete populations e.g., China [16], Spain [17] and Poland [18]. Excluding some internal reliability issues within some subscales (e.g., moderate scores for LTF and AOE factors), the construct validity of the refined/adapted versions were deemed acceptable. However, to date, all studies that have sought to refine the TDEQ have been conducted in populations within developed countries (United Kingdom, Singapore, China, Spain, Poland) where sport development challenges may be less pronounced. Given the accepted complexity and culturally specific nature of talent development; the underlying constructs of the current (English) version of the TDEQ-5 may not be as relevant nor applicable within the Caribbean context, and as such, it is recommended that its psychometric properties be examined within such environments.

Significant benefits to practitioners, stakeholders and researchers have been derived from the use of the TDEQ in both applied and research sport settings. Within a research context, the TDE of young athletes have been examined in relation to key outcomes for their health, wellbeing and performance e.g., needs satisfaction and burnout [19], achievement goals and life aspirations [15], wellbeing [20], and mental toughness [21]. For example, Ivarrson and colleagues [20] found that talented youth Swedish footballers who perceived their TDE as supportive and conducive to long-term development, seemed to be less stressed and experienced higher levels of wellbeing. Alternatively, within an applied context, the TDEQ has been used to evaluate the strengths and weaknesses of elite development environments in a bid to help coaches and practitioners gain feedback, structure interventions and evaluate impact [13, 22, 23]. For instance, Mills, Butts et al.’s [22] assessment of the development environments of 50 elite youth football academy players’ revealed key strengths in the coaching, organizational and sport-related support areas, however deficiencies in athlete understanding, links to senior progression and key stakeholder relationships were noted. Subsequent recommendations were put forward by the authors to help address the aforementioned concerns. As such, developing countries stand to benefit significantly from the potential insights that can be gained from the assessment their TDE which may lead to the formulation more evidence-based strategies that can improve sport development pathways and practices.

The aforementioned economic limitations within the Caribbean coupled with the historical lack of resources infused into sport development and research [8] have likely hindered the implementation of effective talent development strategies within the region. As such, increased knowledge and understanding of the key processes, in conjunction with a method by which these processes can be evaluated will enable the broader and coherent application of evidence-based practice for key stakeholders (e.g., formulation and revision of sport development polices as well as the assessment of coach interventions and impact) [12, 13]. The refined TDEQ-5 has been deemed a plausible measure to assess and monitor the development environments of young talented athletes [14], however, to date the scale has only been validated within a limited number of contexts and only within athlete populations of more developed sporting nations [14, 16, 17]. As such, the relevancy and applicability of the instrument may not be transferable to the Caribbean context due to reasons relating to potential cultural idiosyncrasies and/or underdeveloped sport development systems within the region. Therefore, the principal aim of this study was to examine the psychometric properties of the TDEQ-5 to determine its validity to assess the development environments of talented youth track and field athletes in the Caribbean region. As a secondary aim, we sought to examine athletes’ perceptions of their talent development environment within this context.

## Method

### Overview of research population

Within the English-speaking Caribbean region there are no talent development academies. As such, young talented athletes are developed through private track and field clubs or high schools with sport orientations. Eligibility for recruitment in this study required that all participants be selected from the top-tier track and field clubs and ‘sporting’ high schools within their respective countries. The selection of clubs/schools for the recruitment of athletes were based on the top 25 percentile ranking of schools and clubs in the 2017 junior track and field championships within each respective country. Participants belonged to schools and clubs from within either the urban or rural geographical regions.

### Participants

A sample of 400 youth track and field athletes from six English-speaking Caribbean countries (i.e., Jamaica, Trinidad and Tobago, St Kitts and Nevis, Antigua and Barbuda, Dominica, and Curacao) were recruited for this study. Participants fell within the age range of 13 and 20 years. The clubs and schools were selected through purposive sampling of the top tier track and field clubs and schools via the industry connections of the first author. Of the 400 athletes who participated in the study, 40 cases had missing responses, therefore a total of n = 360 participants (53% males and 47% females) were included in the final analysis for the study. Participants were categorized into the following age ranges: 13–15 years = 39%, 16–18 years = 50% and 19–20 years = 11%. Geographically, 67% of the participants lived in urban areas whereas 33% in rural areas. Participants were recruited from five different disciplines within track and field (sprints/hurdles, middle/long-distance, throws, jumps and multi-events). On average, 33% had trained in their sport for ≤ 10 hours per week and 66% > 10 hours per week. Furthermore, 36% of participants trained < five days per week and 64% ≥ five days per week.

### Procedure

Ethical approval for the project was granted by the first author’s university human ethical review board. Informed written consent from participants and their parents/guardians to participate in the study was obtained before conducting the survey. Participants completed the survey using a secure online website which included demographic questions regarding the participants’ age, gender, country of origin, geographical region, hours and days of training per week. Mobile devices containing the survey were distributed to participants in quiet rooms (e.g., classrooms, meeting rooms within sporting facilities) under the supervision of the first author and a pre-selected administrative team. These administrators provided support to the participants as necessary. Participants were encouraged to respond to the survey honestly, and it was emphasized that there were no right or wrong answers. The survey took participants approximately 15 minutes to complete.

### Measures

The 25-item TDEQ-5 [14] along with questions requesting demographic information were used to examine talented youth athletes’ perceived talent development environment experiences. The scale included five factors: (LTF) long-term development focus “e.g., My training is specifically designed to help me develop effectively in the long-term”; (HQP) holistic quality preparation e.g., “My coach rarely talks to me about by wellbeing”; (SN) support network e.g., “I can pop to see my coach or other support staff whenever I need to”; (COM) communication e.g., “My coach and I often try to identify what my next big test will be before it happens”and, (AOE) alignment of expectations e.g., “I regularly set goals with my coach that are specific to my development”. A six-point Likert scale (1 = “strongly disagree”, 6 = “strongly agree”) was used to measure participant responses. Adequate convergent validity and discriminant validity of the TDEQ-5 has been reported [14]. Furthermore, adequate internal reliability within all five subscales was also reported in talented Singaporean youth athletes (α = .79 - .86) [14].

### Data analysis

Scores from negatively worded items were reversed before data analyses and all statistical analyses were conducted using Stata/SE 15.0 (StataCorp,TX). Before conducting the main analysis, preliminary analyses for missing data were conducted. Missing data was initially imputed using the Expectation-Maximization algorithm [24] for the 40 cases where there was incomplete data. This imputation method is considered acceptable if a proportion of missing values is less than five percent [25]. However, the results showed a reduced global model fit for n = 400. The authors therefore decided to not proceed with the imputation of the incomplete data which resulted in 40 cases with missing values excluded. Subscale scores were reported as means and standard deviations.

For the main analysis, confirmatory factor analyses were conducted to test the factorial validity of the TDEQ-5. A sample size of 200 or above, with a subject to item ratio of at least ten to one is typically deemed adequate for confirmatory factor analysis [25]. The five-factor model with items loading on the following subscales were computed and subsequently inter-correlated: LTF–five items (questions 1–5); AOE–five items (questions 6–10); COM–four items (questions 11–14); HQP–seven items (questions (15–21) and SN–four items (questions 22–25). Factor loading estimates provide an indication of the item level of convergent validity, which should be higher than 0.5 and ideally greater than 0.7 [25, 26]. Discriminant validity is considered present when the 95% confidence interval of estimated correlations between the two latent factors does not include 1.00 [27].

Multiple fit indices were used to assess the global model fit: *X*^2^ to degree of freedom ratio (*X*^2^/*df*), Comparative Fit Index (CFI), Root Mean Square Error of Approximation (RMSEA) with 90% confidence interval (90% CI), the Tucker-Lewis Index (TLI) and the Standardized Root Mean Square Residual (SRMR). Traditional cut-off values of CFI and TLI higher than .90 and RMSEA and SRMR values below .08 were applied as indicators of an acceptable fit [28, 29]. The internal reliability and inter-factor correlations of the TDEQ subscales were computed. To assess internal model fit, composite reliability for the factors and Cronbach’s alpha (α*)* for the subscale scores were used. Values of .70 or above for composite reliability and α indicate adequate reliability [25]. Overall and internal model fit tests are unable to provide information about the reasons of model misfit, and therefore modification indices were used to identify focal areas of ill fit [30]. As the modification index is sensitive to sample size, the standardized expected parameter change was considered in tandem with the index to determine if it was necessary to re-specify the model [30]. In addition to taking references to the modification index and expected parameter change, model re-specification was made only when there was substantive theory to support it [31].

As recommended by Martindale and colleagues [32], mean scores were analyzed and reported at both the subscale and item levels to examine athletes’ perceptions of their TDE in an applied context. All items were subsequently quartile ranked by proportion of agreement to determine the key strengths and areas for improvement as perceived by the athletes. Items ranked in the top quartile (i.e., top 25^th^ percentile) had greater than 84% proportion of agreement and were therefore classified as strengths of the development environment. Conversely, items ranked in the bottom quartile (i.e., bottom 25^th^ percentile) had less than 65% proportion of agreement and were subsequently categorized as areas for improvement within the environment.

## Results

### Goodness-of-fit

The authors of this study sought to develop the most concise and best-fitting model, however, the CFA model with 25 items did not meet all of the thresholds of good fit. The model showed good fit based on RMSEA (.050) and SRMR (.055) values, however the CFI (.875) and TLI (.859) values were slightly below the acceptable .90 value indicating adequate fit (see Table 1). Several items had relatively weak standardized loadings, primarily on the LTF and AOE subscales (see Table 2). Inspection of modification indices indicated that adding covariances between error terms for items AOE1 and AOE2 (*X*^2^ = 15.4, expected parameter change = .363), SN1 and SN2 (*X*^2^ = 17.3, expected parameter change = .438) AOE2 and SN4 (*X*^2^ = 15.8, expected parameter change = .336), AOE5 and COM1 (*X*^2^ = 16.4 expected parameter change = .276) and AOE1 and HQP2 (*X*^2^ = 13.5, expected parameter change = .336) would have relatively large impacts on improving model fit. In each of the aforementioned cases, the items measured contents that were either related to both subscales and/or showed a relationship within subscale between items that was outside of the scope of the respective subscale. As a result, it was considered that correlating their errors was theoretically appropriate. The measurement model was therefore re-specified with correlations added between the above-mentioned five pairs of error terms. The model fit of the re-specified model was improved over the originally specified model: CFI (.915), TLI (.902), RMSEA (.042), 90% CI (.034, .049), SRMR (.052) (see Table 1).

**Table 1:** Goodness-of-fit statistics for TDEQ-5 and re-specified TDEQ-5 models.

	df	*X*^2^	*p*	CFI	TLI	RMSEA	SRMR
**TDEQ-5 CFA**	265	505	< .001**	.875	.859	.050	.055
**Re-specified TDEQ-5 CFA**	260	423	< .001**	.915	.902	.042	.052

** p < .001.

X^2^: Chi-square_ms; df: degree of freedom; CFI; comparative fit index; TLI: Tucker-Lewis index; RMSEA: root mean square error of approximation; SRMR; standardized root mean square residual; p: p-value.

### Convergent and discriminant validity

Factor loading estimates indicated a satisfactory level of convergent validity for three subscales (COM, HQP and SN) with all factor loadings higher than the recommended .50 except for one item (HQP1: .43). However, factor loadings within the LTF and AOE subscales were all below .50 and ranged from (.22 to .49) except for 1 item (AOE5: .60; see Table 2). As expected, the correlations between subscales were all positive (r = .17 - .51; see Table 3). The latent factor correlations ranged from .34 - .95 with only the LTF/AOE correlation including 1.00 with the following 95% confidence interval (CI) (.78–1.12), thus supporting only partial discriminant validity and convergent validity of the TDEQ-5 scale across three subscales (COM, HQP and SN).

**Table 2:** Factor loadings for the TDEQ-5 and re-specified TDEQ-5 models.

SUBSCALES	ITEMS	TDEQ- 5 MODEL	TDEQ- 5 MODEL Re-specified
**LTF**	1	.40	.39
	2	.39	.39
	3	.42	.43
	4	.35	.34
	5	.22	.22
**AOE**	6	.54	.49
	7	.40	.36
	8	.46	.46
	9	.28	.30
	10	.62	.60
**COM**	11	.68	.65
	12	.63	.64
	13	.72	.74
	14	.51	.51
**HQP**	15	.43	.43
	16	.54	.53
	17	.51	.51
	18	.66	.66
	19	.74	.74
	20	.53	.53
	21	.63	.64
**SN**	22	.56	.50
	23	.68	.63
	24	.83	.87
	25	.61	.60

Note: TDEQ– 5 factor Talent Development Environment Questionnaire: LTF–long-term development focus; AOE–alignment of expectations; COM–communication; HQP–holistic quality preparation; SN–support network.

**Table 3:** Descriptive statistics, reliability, and correlations among studied variables (n = 360).

	CR	α	M	SD	LTF	AOE	COM	HQP	SN
**1. LTF**	.42	.42	4.83	0.62	-	.95	.62	.35	41
**2. AOE**	.55	.57	4.50	0.81	.45	-	.81	.45	.57
**3. COM**	.73	.73	4.70	0.95	.33	.51	-	.42	.56
**4. HQP**	.78	.78	4.19	1.00	.17	.29	.35	-	.34
**5. SN**	.75	.77	3.78	1.10	.20	.38	.37	.21	-

CR: composite reliability; LTF: long-term development focus; AOE: alignment of expectations; COM: communication; HQP: holistic quality preparation; SN: support network. The latent factor correlations are presented above the diagonal, the zero-order correlations between subscales are presented below the diagonal. P < .01 for all factor/subscale correlations

### Internal reliability and composite reliability

Table 3 presents the results of the internal reliability, composite reliability and latent factor correlation matrix of the re-specified TDEQ-5 model. Cronbach alpha for the TDEQ-5 scale was α = .83, which was deemed acceptable. However, when considering the individual subscales, both the LTF (.42) and AOE (.57) subscales had moderate to low internal consistency, below the acceptable .70 value (see Table 3). Internal consistency for the COM (.73), HQP (.77) and SN (.77) subscale scores was deemed acceptable. Levels of composite reliability were generally similar to internal reliability.

### Descriptive statistics (subscale and item level)

Table 3 shows the mean and standard deviations of the subscale scores of the main variables highlighting athletes’ overall factor level perceptions of their development environment. Participants reported moderate (3.78) to high (4.83) scores of the five TDE factors. However, the LTF and AOE factors showed low internal reliability (α = .42 and .57) respectively, and were subsequently omitted for interpretation at the factor level. Overall, athletes perceived their development environment as positive and supportive highlighting good coach-athlete communication at the youth level. Notwithstanding, athletes’ perceived their environment as lacking holistic quality preparation and sport science/medicine support. Table 4 displays the mean and standard deviations of the item level scores for all five TDEQ factors in order to provide a deeper and more meaningful understanding of athletes perceptions beyond the subscale scores. At an item level, six key strengths (light grey) and six areas for improvement (dark grey) within the environment emerged from the data set in line with the top and bottom 25^th^ percentile categories (see Table 4). Factors are discussed below from strongest to weakest at a subscale and item level where applicable.

**Table 4:** Means and standard deviations for TDEQ items within each factor.

ITEM #	TDEQ STATEMENTS	MEAN	*SD*
1	My training is specifically designed to help me develop effectively in the long-term	5.27	0.89
14	My coach explains how my training and competition programme work together to help me develop	5.08	1.11
3	I spend most of my time developing skills and attributes that my coach tells me I will need if I am to compete successfully at the professional level	4.95	0.99
2	My coach emphasizes that what I do in training and competition is far more important than winning	4.94	1.23
11	My coach and I regularly talk about things I need to do to progress to the top level in my sport	4.85	1.30
4	My coach allows me to learn through making my own mistakes	4.69	1.28
7	The advice my parents give me fits well with the advice I get from my coaches	4.63	1.44
12	My coach and I talk about what current and/or past world-class performers did to be successful	4.62	1.31
9	I am involved in most decisions about my sport development	4.59	1.24
8	My progress and personal performance is reviewed regularly on an individual basis	4.54	1.13
10	I regularly set goals with my coach that are specific to my individual development	4.46	1.40
16	My coach does not appear to be that interested in my life outside of sport	4.43	1.60
17	My coach rarely takes the time to talk to other coaches who work with me	4.40	1.47
5	I would be given good opportunities even if I experienced a dip in performance	4.29	1.24
6	My coaches make time to talk to my parents about me and what I am trying to achieve	4.28	1.41
21	I am not taught that much about how to balance training, competition and recovery	4.26	1.61
25	Those who help me in my sport seem to be on the same wavelength as each other when it comes to what is best for me	4.26	1.42
13	My coach and I often try to identify what my next big test will be before it happens	4.25	1.35
20	The guidelines in my sport regarding what I need to do to progress are not very clear	4.21	1.43
15	My coach rarely talks to me about my wellbeing	4.16	1.60
18	I don’t get much help to develop my mental toughness in sport effectively	4.07	1.61
19	I am rarely encouraged to plan for how I would deal with things that might go wrong	3.81	1.57
23	I can pop in to see my coach or other support staff whenever I need to	3.79	1.58
24	My coaches talk regularly to the other people who support me in my sport about what I am trying to achieve	3.75	1.52
22	Currently I have access to a variety of different types of professionals to help my sports development	3.31	1.62

Strengths (light grey Rows) and areas for improvement (dark grey Rows) of Caribbean TDE as perceived by youth track and field athletes.

#### Long-term development focus

This factor comprises five items and entails the extent to which developmental programmes are specifically designed to facilitate athlete’s long-term success (e.g., fundamental training and rounded development, ongoing opportunities, and de-emphasis of winning) [12]. Due to the subpar alpha coefficient (α = .42), the mean subscale score was not interpreted for this factor, however four items emerged within the top 25^th^ percentile. At an item level, most athletes (96%) felt that their training was specifically designed to help them develop in the long-term (M = 5.27), with great emphasis being placed by the coach on the development of fundamental skills and attributes for long-term success (M = 4.95). A large majority of athletes agreed that their coach cared more about their development rather than them winning (M = 4.94) and allowed them to learn through making their own mistakes (M = 4.69).

#### Communication

This factor comprises four items and evaluates the quality and efficacy of communication between the coach and athlete in both formal and informal settings (e.g., development path, rationale for training, feedback) [12]. This factor scored second highest behind the LTF factor with a mean subscale score of 4.70, and was generally perceived as a strong component of the environment with two factors categorized in the top 25^th^ percentile. Within this subscale, 86% of athletes agreed that they had good communication with their coaches about their progress within the sport (M = 4.85). Approximately 91% of athletes were in agreement with the level of explanation from their coaches’ regarding the interconnections between their training and competition developmental programmes (M = 5.08).

#### Alignment of expectations

This factor consists of five items and entails the extent to which goals for sport development are coherently set and aligned (e.g., goal setting, goal review, and individualized goals) [12]. Due to the subpar alpha coefficient (α = .57), the mean subscale score was not interpreted for this factor. Overall, the items within this subscale registered moderate scores with no items having been categorized within the top or bottom 25^th^ percentile. At an individual item level, most athletes felt that their coach made time to talk with their parents about the athletes’ goals (M = 4.28), and that the advice received from their parents generally aligned with that of the coaches (M = 4.23). Additionally, athletes generally felt involved in most decisions about their sport development (M = 4.59), with periodic goal setting (M = 4.46) regular performance appraisals being conducted by their coaches (M = 4.54).

#### Holistic quality preparation

This factor comprises seven items and entails the extent to which intervention programmes are prepared both inside and outside of sport settings (e.g., caring coach, clear guidance, mental preparation and balanced life) [12]. This factor registered the second lowest mean score of 4.19 and was viewed as one of the weaker elements of the environment with three items categorized in the bottom 25^th^ percentile. More specifically, key limitations identified by athletes showed that approximately 36% did not perceive themselves to have received adequate help to develop their mental toughness (M = 4.07), with 44% having expressed that they were rarely encouraged to plan for how to deal with things that might go wrong (M = 3.81). Approximately 35% of athletes expressed that their coach rarely spoke to them about their wellbeing (M = 4.16).

#### Support network

This factor comprises four items and refers to the extent to which a coherent, approachable and wide-ranging support network is available for the athlete (i.e., physiotherapist, sport psychologist, strength trainer, nutritionist, lifestyle advisor) [12]. This factor had a mean score of 3.78 and was considered the lowest performing component of the environment with three items categorized in the bottom 25^th^ percentile. Within this factor, 53% of athletes expressed that they felt that they did not have adequate access to a variety of sport science/sport medicine professionals to help with their sports development (M = 3.31). Athletes reported that their coach did not regularly talk with support staff about what they were trying to achieve (M = 3.75), but nevertheless generally considered their coaches and support personnel to be on the same wavelength regarding the athletes’ best interest (M = 4.26).

## Discussion

The process of developing young athletes into elite senior athletes needs to be multidimensional and should assess all aspects of the coaching environment [33]. To date, there has been a growing interest in talent development research within both the applied and research context that has explored the development environments of youth athlete populations within primarily more advanced sporting nations [13,15, 19, 20, 21, 22, 23]. However, limited research has been conducted on athlete populations within developing nations, and none within the Caribbean context. Caribbean countries stand to benefit significantly from the potential insights that can be gained from the assessment of their development environments which can lead to the broader and coherent application of evidence-based practices by coaches and administrators within the region. The TDEQ scale provides a quantifiable evidence-based approach to examine the development environment and its impact on the developing athlete [12, 13], however its validity and applicability is unknown within the Caribbean. Consequently, extending the literature, this study sought to examine the underlying factor structure of the TDEQ-5 instrument to determine its validity for use within the youth athlete populations of the English-speaking Caribbean region. A subsequent preliminary examination of athletes’ perception of their TDE within this context was undertaken. The main finding of this study revealed that the confirmatory factor analysis confirmed the re-specified five-factor structure showing adequate global model fit, notwithstanding some limitations noted within two of its constructs.

A key finding for this study showed that the re-specified TDEQ-5 model had adequate global model fit. Model re-specification with added co-variances between specified error terms was made only when there was substantive theory to support it [31]. Plausible justifications for item correlations are discussed as follows. Firstly, a close examination of items AOE1 (“My coaches make time to talk to my parents about me and what I am trying to achieve”) and AOE2 (“The advice my parents give me fits well with the advice I get from my coaches”) revealed that these items seemed to additionally assess communication with or about the athlete from their social support network (i.e., parents, coaches). Secondly, items SN1 (“Currently, I have access to a variety of different types of professionals to help my sports development”) and SN2 (“I can pop in to see my coach or other support staff whenever I need to”) both assessed the level of access that athletes have to their support staff, and therefore was deemed as correlated. Thirdly, item AOE2 (“The advice my parents give me fits well with the advice I get from my coaches”) and SN4 (“Those who help me in my sport seem to be on the same wavelength as each other when it comes to what is best for me”) were deemed as correlated as both entailed the alignment of expectations between the athletes’ social support staff. Fourthly, item AOE5 (“I regularly set goals with my coach that are specific to my individual development”) and COM1 (“My coach and I regularly talk about things I need to do to progress to the top level in my sport”) both seek to assess the degree of communication shared between athlete and coach. Lastly, items AOE1 (“The advice my parents give me fits well with the advice I get from my coaches”) and HQP2 (“My coach does not seem to be interested in my life outside of sport”) were deemed to be correlated as both items touched on the level of interest or guidance provided by the coach as perceived by the athlete.

Adequate convergent and discriminant validity was found within three of the of the TDEQ-5 subscales (i.e., COM, HQP and SN), but not for the LTF and AOE subscales. Findings were partially consistent with previously published research that have confirmed the validity of the scale in young talented athletes [14, 16, 17]. In fact, acceptable construct validity has been reported within both the English and Chinese versions of the TDEQ-5 [14, 16]. Similar findings of acceptable convergent and discriminant validity were found in the Spanish version of the TDEQ-5 within 322 regional level athletes [17]. One possible reason for the low construct validity within the two reported subscales in this study is that the individual items may not have accurately measured the underlying construct as suggested [34], specifically within this athlete population. Furthermore, one plausible reason for the only low factor loading within the holistic quality preparation subscale; item HQP1 (“My coach rarely talks to me about my well-being”) may be linked to some degree of misunderstanding by participants. HQP1 was the first item within the scale that was negatively worded which may have resulted in a higher degree of misresponse by participants (i.e., an individual selects an answer that is opposite to his/her perceptions) and contributed to the low factor loading. It has been reported that reversed items can be more confusing for respondents and that substantial difficulty may arise during item judgment which increases misresponse [14, 35]; and may be even more prevalent within young participants [36].

Similar to the aforementioned construct validity findings, internal reliability was found to be adequate within three of the of the TDEQ-5 subscales (i.e., COM, HQP and SN) but subpar within two of the subscales (i.e., LTF, and AOE). The relatively low observed internal reliability of the LTF and AOE subscales of the TDEQ-5 were similar to findings reported in some previous studies on young talented athletes within Spain (LTF α = -.62 and AOE α = -.62) [17], China (LTF α = -.68) [16] and Poland (LTF α = .66) [18]. In contrast, adequate internal reliability within all five subscales has been reported in talented youth athletes within Singapore (α = .79 - .86) [14]. Notably, the LTF and AOE subscales were the first 10 items included within the survey for this study. Another plausible explanation for the lower internal reliability scores within these two subscales may be that participants may not have initially comprehended the item content at the beginning of the survey. This may have resulted in some meaning loss and increased measurement error across both subscales. As such, the scores may be applying different standards when evaluating the participants’ responses [34]. For instance, the items included in the long-term development focus and alignment of expectation subscales may have a different connotation of sport development for Caribbean athletes when compared to more westernised athlete populations. Historically there has been limited long-term planning and policy formation and investment in sport within the Caribbean region [8, 9] which can translate into an athlete development pathway that is opaque and not fully understood by the athletes within it.

### Strengths and areas for improvement

Caribbean athletes generally perceived their TDE as positive and supportive which was consistent with other studies on talented youth athletes from more developed sporting nations [16, 19]. Some of the key strengths highlighted emerged from the long-term development focus factor where coaches were perceived to have placed significant emphasis on the longer-term athletic development outcomes of athletes. Evidence from within the talent development literature have corroborated this approach towards young athletes’ development in that greater emphasis is placed on the prescription of appropriate training programs commensurate with young athletes’ technical ability and stage of development [37]. In fact, to maximize long-term success of young athletes, the National Strength and Conditioning Association (NSCA) Position Statement recommends the primary focus be on early sampling, motor skill and muscular development with health and wellbeing being the central tenet of such programs [38]. Other key emergent strengths from this study highlighted the efficacy of the coach-athlete communications regarding the athletes’ development. The impact of autonomy supportive coach behaviors in young athlete’s development have been shown to lead to increased intrinsic motivation, enjoyment, interest in activities and successful competitive performance [39, 40]. Good communication between coaches and athletes have also been shown to promote a great deal of understanding and in turn an even more stable, harmonious and healthy coach–athlete relationships [40, 41].

Notwithstanding these strengths, comparatively lower scores were registered for the holistic quality preparation and support network factors. The limitations noted at the item level revealed inadequate support for athletes’ wellbeing, development of their mental toughness and coping skills as well as a general lack of access to sport science/medicine support. Inaccessibility to the aforementioned support may be more pronounced within the Caribbean as there are no talent development academies within the region [42]. Therefore, athletes classified as junior-elite are not placed within specialized talent development academies or schools but remain part of the larger group of developing athletes, thus potentially limiting more individualized support. In more advanced sporting nations, talent development is more systematic and individualized and is conducted through specialized organizations like the child and youth sport schools in East European countries (e.g., Russia) or national talent search programs at the Australian Institute of Sport (https://ais.gov.au), and the UK high performance talent program at UK Sport (https://www.uksport.gov.uk/our-work/talent-id) [43]. A lack of a clear and cohesive plan and strategy within the Caribbean context is therefore more evident when juxtaposed with the talent development processes undertaken within more advanced sporting nations [44, 45]. As such, a higher quality TDE within the region may be achieved by improving the quality of preparation of athletes by improving the accessibility and availability of individualized sport science/medicine related support at the youth elite level.

### Limitations

Some limitations of the study should be taken into consideration when interpreting the findings. Firstly, even though participants from this study were selected from within the top 25 percentile ranking of schools and clubs in the 2017 junior track and field championships, it is possible that not all athletes within this study were highly trained (i.e., 33% had trained ≤ 10 hours per week). As such, some caution should be taken when generalizing findings to other contexts. Additionally, there also may have been some lack of understanding of specific items within the survey resulting in the subpar validity and reliability scores within the LTF and AOE subscales and misresponse in the HQP1 item. It is therefore important for future researchers to remind participants to take care when reading the questions and make sure they understand what is being asked, so as to avoid careless responses to items. Finally, although the TDEQ-5 has been developed as a generic tool assessing environmental factors across sport, stage of talent development, and culture, there will be context-specific requirements within talent development that are not included within the current scale [12].

### Implications and recommendations

It is recommended that future researchers investigating the TDE in Caribbean contexts, examine the content validity of the LTF and AOE subscales within this athlete population and provide further psychometric evidence of the scale such as test-retest reliability, concurrent validity and criterion validity. Furthermore, the re-specified TDEQ-5 may be used to conduct future research on Caribbean athletes in order to understand and unpack how the environment is related to important outcomes e.g., motivation [15], stress and wellbeing [20], burnout [19], mental toughness [21]. In an applied research context, it is recommended that practitioners use the TDEQ-5 instrument in its current form on an item by item basis to evaluate the strengths and weaknesses of different talent development environments within the region to help administrators and coaches gain feedback, structure interventions, and evaluate impact across various sports [13, 22]. However, it is imperative that item wording and meaning is clearly explained to athletes within context (more so for the LTF and AOE items) to minimize meaning loss and misinterpretation of information. The findings in this study highlight six key areas for improvement within the SN and HQP subscales that can be employed by stakeholders involved in talent development programmes in the Caribbean region to facilitate better athlete preparation and support. Youth elite athletes would benefit from the formulation of a national ‘merit’ based and tiered support services program to allow access to state funded sport science and sport medicine services. This program should ideally be run through a national sport organization with specific responsibilities for elite sport (e.g., national sport institute) to allow for effective delivery of services to athletes.

## Conclusions

The findings in this study provide preliminary support for the use of the re-specified TDEQ-5 within the Caribbean context as evidenced by the scores suggesting adequate model fit of a re-specified model and good overall reliability of the scale. Moreover, the TDEQ-5 instrument showed adequate construct validity and internal reliability within three subscales (COM, HQP and SN) although the validity and reliability within two subscales (LTF and AOE) were below average. It is therefore recommended that the scale be used with some caution in both applied and research settings when interpreting the results of the LTF and AOE subscales within the Caribbean context. Preliminary findings on athletes’ perceptions of their TDE revealed key strengths in coaches’ long-term development focus and communications however deficiencies were noted in the accessibility and availability of sport-related support and preparation of athletes. Further scale development and testing using both quantitative and qualitative methodological approaches to determine the appropriate cultural context for items and wording within the LTF and AOE subscales may be necessary.
